# Hospital admissions and mortality over 20 years in community-dwelling older people: findings from the Hertfordshire Cohort Study

**DOI:** 10.1007/s40520-023-02554-0

**Published:** 2023-09-13

**Authors:** Roshan Rambukwella, Leo D. Westbury, Camille Pearse, Kate A. Ward, Cyrus Cooper, Elaine M. Dennison

**Affiliations:** 1 MRC Lifecourse Epidemiology Centre, University of Southampton, Southampton, UK; 2 NIHR Southampton Biomedical Research Centre, University of Southampton and University Hospital Southampton NHS Foundation Trust, Southampton, UK; 3 NIHR Oxford Biomedical Research Centre, University of Oxford, Oxford, UK; 4 Victoria University of Wellington, Wellington, New Zealand

**Keywords:** Ageing, Hospitalisation, Mortality, Epidemiology, Risk factor

## Abstract

**Background:**

Demographic changes worldwide are leading to pressures on health services, with hospital admissions representing an important contributor. Here, we report admission types experienced by older people and examine baseline risk factors for subsequent admission/death, from the community-based Hertfordshire Cohort Study.

**Methods:**

2997 participants (1418 women) completed a baseline questionnaire and clinic visit to characterize their health. Participants were followed up from baseline (1998–2004, aged 59–73 years) until December 2018 using UK Hospital Episode Statistics and mortality data, which report clinical outcomes using ICD-10 coding. Baseline characteristics in relation to the risk of admission/death during follow-up were examined using sex-stratified univariate logistic regression.

**Results:**

During follow-up, 36% of men and 26% of women died and 93% of men and 92% of women had at least one hospital admission; 6% of men and 7% of women had no admissions and were alive at end of follow-up. The most common types of admission during follow-up were cardiovascular (ever experienced: men 71%, women 68%) and respiratory (men 40%, women 34%). In both sexes, baseline risk factors that were associated (*p* < 0.05) with admission/death during follow-up were older age, poorer SF-36 physical function, and poorer self-rated health. In men, manual social class and a history of smoking, and in women, higher BMI, not owning one’s home, and a minor trauma fracture since age 45, were also risk factors for admission/death.

**Conclusions:**

Sociodemographic factors were related to increased risk of admission/death but a small proportion experienced no admissions during this period, suggesting that healthy ageing is achievable.

**Supplementary Information:**

The online version contains supplementary material available at 10.1007/s40520-023-02554-0.

## Introduction

Demographic changes associated with an ageing population are associated with higher usage of health resources and require careful consideration to provide sustainable health services in the future. In the United Kingdom, the number of people aged 60 and over is projected to increase from 14.9 million in 2014 to 21.9 million by 2039 [1]. As part of this growth, the number of over-85s is estimated to more than double from 1.5 million in 2014 to 3.6 million by 2039 [2]. In the UK, two-thirds of people admitted to hospitals are over 65 years old, and those over 85 account for 25% of bed days [2]. As a consequence, well-developed health systems such as in the UK are struggling as it has been shown that despite the relatively rapid increase in life expectancy over the years, healthy life expectancy has increased slowly, resulting in more burden on health services [3]. Although increasing age is associated with increased clinical service usage, other factors may also play a substantial role. For example, multimorbidity and socioeconomic deprivation are associated with increased admission risk [4, 5].

The Hertfordshire Cohort Study (HCS) is a population of UK community-dwelling participants [6]. Previously, we have linked the data from HCS to Hospital Episode Statistics (HES) data to obtain information on hospital admissions [7]. Our results at that time showed that 68% of the participants had at least one hospital admission during the follow-up period (median follow-up of 10 years). At that time, the most common reasons for admission were circulatory diseases (31%), injuries and poisonings (14%), and respiratory diseases (13%). Here, we update this study to report on 20 years of follow-up, including medical and socioeconomic risk factors for hospital admission or death. In this analysis, we were particularly interested to identify how admission types have changed over time from the previously reported analysis of admissions, and to examine what proportion of our cohort members had avoided hospital admissions altogether.

## Methods

### The Hertfordshire Cohort Study

The Hertfordshire Cohort Study (HCS) comprises 2997 men and women born in Hertfordshire from 1931 to 1939 and who still lived there from 1998 to 2004 when they completed a home interview and clinic visit for a health assessment. The HCS had ethical approval from the Hertfordshire and Bedfordshire Local Research Ethics Committee and all participants provided written informed consent for the investigations they underwent in the clinic and for researchers to access their medical records in the future. Further details of HCS have been described previously [6, 7]. The HCS is one of the few studies in the UK which is linked to HES data.

### Ascertainment of participant information in 1998–2004

Smoking status, weekly alcohol consumption and physical activity (Dallosso questionnaire) were ascertained by a nurse-administered questionnaire at the home interview [8]. Participants completed a food-frequency questionnaire from which a ‘prudent diet’ score was derived using principal components analysis; higher scores reflected healthier diets. Occupational social class was ascertained from the most recent or current full-time occupation for men and among women who never married, and from husband’s occupation for ever-married women. Occupations were then classified according to the 1990 OPCS Standard Occupational Classification (SOC90) unit group for occupation [9]. Information on housing tenure (owned/mortgaged versus rented/other) was also collected. Self-rated general health was ascertained from this item of the SF-36 Health Survey. This required participants to select one of the following options to describe their health: ‘Excellent’; ‘Very good’; ‘Good’; ‘Fair’; or ‘Poor’. Self-reported physical function was assessed according to 10 questions from the physical functioning scale of the SF-36 Health Survey [10]. Each question was scored such that the total physical function score could range from 0 to 100; participants with scores in the bottom sex-specific fifth of the distribution (≤ 75 for men, ≤ 60 for women) were classified as having a poor physical function as in previous analyses [11]. Self-reported walking speed was ascertained by asking participants which of the following responses best describes their walking speed: ‘unable to walk’; ‘very slow’; ‘stroll at an easy pace’; ‘normal speed’; ‘fairly brisk’; or ‘fast’. Fracture history since age 45 years was also ascertained; fractures were categorized as major trauma or minor trauma.

At the baseline clinic, height was measured (Harpenden pocket stadiometer, Chasmors Ltd, London, UK) along with weight (SECA floor scale, Chasmors Ltd, London, UK) and used to derive BMI. Grip strength was assessed three times for each hand using a Jamar dynamometer; all analysis used the highest grip strength measurement.

### Ascertainment of adverse health-related events

Adverse health events were identified using mortality and Hospital Episode Statistics (HES) data. Permission to obtain a HES extract for HCS participants from 01/04/1998 to 31/12/2018 was granted by the Ethics and Confidentiality Committee of the National Information Governance Board and NHS Digital. The Linkage of the HCS cohort with HES data has been previously described [7]; the HES extract included admission information such as the admission date, diagnoses coded to ICD-10, and date of discharge. Adverse health-related events were identified by admissions assigned with ICD-10 codes as stated in Supplementary Table 1.

### Statistical methods

Participant characteristics at baseline and adverse health events experienced during follow-up were described using summary statistics. Baseline characteristics in relation to risk of admission/death during follow-up were examined using sex-stratified logistic regression; sex-specific standard deviation scores (*z*-scores) were derived for all continuous exposures to enable comparison of effect sizes. All analyses were stratified by sex; *p* < 0.05 was regarded as statistically significant. Analyses were conducted using Stata, release 17.0.

## Results

### Adverse events experienced by participants during follow-up

Summary statistics for the participant characteristics at baseline and adverse health events during follow-up are presented in Table 1. Mean (SD) age at baseline was 65.7 (2.9) and 66.6 (2.7) years among men and women, respectively. During follow-up, 36% of men and 26% of women died and 93% of men and 92% of women had at least one hospital admission; only 6% of men and 7% of women had no admissions during follow-up and were alive at the end of follow-up.

**Table 1:** Baseline participant characteristics and adverse health events during follow-up

Participant characteristic [mean (SD), median (lower quartile, upper quartile), or %]	Men (*n* = 1579)	Women (*n* = 1418)
Characteristics at baseline (1998–2004)		
Age (years)	65.7 (2.9)	66.6 (2.7)
Height (cm)	174.2 (6.5)	160.8 (5.9)
Weight (kg)	82.4 (12.7)	71.4 (13.4)
BMI (kg/m^2^)	27.2 (3.8)	27.6 (4.9)
Ever smoked regularly	67%	39%
High alcohol intake (units per week: > 21 men, > 14 women)	22%	5%
Prudent diet score	-0.6 (2.1)	0.7 (1.7)
Dallosso physical activity score	60.9 (15.3)	59.0 (15.7)
Social class (manual)	59%	58%
Home ownership (not owned or mortgaged)	19%	22%
Grip strength (kg)	44.0 (7.5)	26.5 (5.8)
SF-36 physical function score	90.0 (80.0, 95.0)	85.0 (65.0, 95.0)
Self-reported walking speed		
Fast	4%	6%
Fairly brisk	27%	22%
Normal speed	40%	45%
Stroll at easy pace	24%	20%
Very slow/unable to walk	5%	7%
Minor trauma fracture since age 45 years	7%	18%
Self-rated health		
Excellent	13%	8%
Very good	38%	32%
Good	38%	44%
Fair	10%	14%
Poor	1%	1%
Events during follow-up (ever had)		
Death	36%	26%
Hospital admission	93%	92%
Death/hospital admission	94%	93%
Types of admission during follow-up (ever had)		
Neurological	23%	20%
Cardiovascular	71%	68%
Myocardial infarction	8%	5%
Stroke	7%	5%
Respiratory	40%	34%
Any fracture	9%	22%
Hip fracture	2%	5%
Fall	13%	21%

Follow-up period lasted from baseline (1998–2004) until 31st December 2018

The most common types of admission during follow-up were cardiovascular (ever experienced: men 71%, women 68%) and respiratory (men 40%, women 34%). Falls (men 13%, women 21%) and fractures (men 9%, women 22%) were more common among women than men.

### Baseline participant characteristics in relation to risk of admission/death during follow-up

Odds ratios for having admissions or dying before the end of follow-up according to baseline participant characteristics are presented in Table 2. Among men, the following characteristics recorded at baseline were related to increased risk of subsequent admission/death: older age (*p* = 0.041); ever smoking (*p* = 0.039); manual social class (*p* = 0.020); lower SF-36 physical function score (*p* < 0.001); and poorer self-rated health (*p* < 0.001). Similar to men, older age (*p* = 0.001), lower SF-36 score (*p* < 0.001), and poorer self-rated health (p < 0.001) were risk factors for admission/death among women. However, additional risk factors for admission/death among women included greater BMI at baseline (*p* = 0.002); lower physical activity (*p* = 0.004); not owner-occupying one’s home (*p* = 0.028); lower grip strength (*p* = 0.002); slower self-reported walking speed (*p* < 0.001); and minor trauma fracture since age 45 years (*p* = 0.012).

**Table 2:** Odds ratios for having admissions or dying before the end of follow-up according to baseline participant characteristics

Participant characteristic	Men		Women	
	Odds ratio (95% CI)	*p* value	Odds ratio (95% CI)	*p* value
Age (*z*-score)	**1.26 (1.01,1.56)**	**0.041**	**1.42 (1.15,1.76)**	**0.001**
BMI (*z*-score)	1.15 (0.92,1.44)	0.219	**1.44 (1.14,1.81)**	**0.002**
Ever smoked regularly	**1.58 (1.02,2.44)**	**0.039**	1.24 (0.81,1.90)	0.327
High alcohol intake (units per week: > 21 men, > 14 women)	0.81 (0.50,1.34)	0.421	0.94 (0.37,2.40)	0.905
Prudent diet score (*z*-score)	0.95 (0.77,1.18)	0.638	1.07 (0.88,1.32)	0.490
Dallosso physical activity score (*z*-score)	0.87 (0.70,1.09)	0.228	**0.73 (0.58,0.90)**	**0.004**
Social class (manual)	**1.68 (1.09,2.61)**	**0.020**	1.13 (0.75,1.70)	0.565
Home ownership (not owned or mortgaged)	1.68 (0.88,3.21)	0.113	**1.95 (1.07,3.55)**	**0.028**
Grip strength (*z*-score)	0.89 (0.71,1.10)	0.275	**0.70 (0.56,0.87)**	**0.002**
SF-36 physical function score (*z*-score)	**0.39 (0.24,0.62)**	**< 0.001**	**0.33 (0.22,0.49)**	**< 0.001**
Self-reported walking speed (per lower band)	1.25 (0.99,1.57)	0.062	**1.53 (1.23,1.91)**	**< 0.001**
Minor trauma fracture since age 45 years	1.65 (0.59,4.59)	0.335	**2.58 (1.23,5.38)**	**0.012**
Self-rated health (per lower band)	**1.65 (1.27,2.13)**	**< 0.001**	**1.62 (1.28,2.07)**	**< 0.001**

For binary exposures, odds ratios are presented for the presence versus absence of the exposure

Sex-specific *z*-scores were coded for all continuous exposures

Significant associations (*p* < 0.05) are highlighted in bold

## Discussion

Most studies on hospital usage in the UK are based on statistics at the hospital or area level. Since the Hertfordshire Cohort Study is community-dwelling and was linked to HES data at the individual-level, it enabled us to compare the factors related to admission to NHS hospitals or death. Over a follow-up of around 20 years, 93% of men and 92% of women had at least one hospital admission; these figures were 68% and 70% for men and women, respectively, over the first 10-years of follow-up [7]. With the advancement of the age of the cohort, this increase would be expected. The most common types of admissions over follow-up were those with cardiovascular and respiratory causes. These findings are consistent with our previous description of hospital admission rates in this cohort conducted by Simmonds et al. [7] with 10 years of follow-up of the HCS, suggesting that there have been no changes in the most common types of admission over recent years. However, our findings also highlight the existence of a group with no admissions or deaths in the cohort over follow-up. It suggests the possibility of having good health with low levels of comorbidity, sometimes termed healthy life expectancy, even into older age.

Socioeconomic factors including low education levels, low income, and lifestyle factors like smoking, low physical activity, and poor diets are known to be associated with lower healthy life expectancy [3], and many of these factors were associated with greater risk of admission/death in this study. The factors associated differed between men and women: manual occupational social class was significantly associated with admission/death among men and not owner-occupying one’s home was associated with admission/death among women. In high-income countries, other studies have shown that lower socioeconomic status is commonly associated with higher hospital admission rates in later stages of life and is consistently associated with a greater risk of dying in hospital [12]. Lower socioeconomic status has been shown to be associated with greater hospital admission rates in older people in other studies conducted in the UK [13, 14]. Our results, therefore, support findings from previous studies that lower socioeconomic status is associated with a greater risk of admission/death and further highlight the importance of considering and intervening in socioeconomically deprived areas to reduce health inequalities in later life.

Considering potentially modifiable lifestyle factors, diet quality and alcohol intake at baseline were not significantly associated with hospital admission/death in either men or women, possibly reflecting a healthy participant bias among our cohort members. In women but not men, higher BMI was significantly associated with admission/death. BMI above the healthy range has been shown to be strongly associated with higher rates of hospital admissions in the UK for both men and women over 60 years in other studies [15] and it is, therefore, possible that we failed to observe an association in men due to lack of statistical power. Ever-smoking for men was significantly associated with admission/death and this finding is consistent with previous studies in similar cohorts in the UK [13].

Self-reported health measures at baseline were associated with outcomes over follow-up. Poor self-rated health was identified as a risk factor for admission/death in the current study. There is a growing body of literature demonstrating a link between poorer self-rated health and higher hospital admission rates among older people in the UK [16]. Poor self-rated health may reflect high comorbidity burden, social isolation, limited mobility, poor nutrition, and low level of physical activity [17]. Likewise, lower SF-36 physical function scores (in men and women) and lower self-reported walking speed (in women only) were related to increased risk of admission/death in our study.

Lower grip strength and physical activity were related to increased risk of admission/death in this study among women; among men, directions of association were the same but did not reach statistical significance. Similarly, lower grip strength was related to greater risk of hospital admission in the previous follow-up of this cohort until March 2010 [18]. Our findings regarding physical activity support the strong evidence base for the beneficial effects of physical activity on health and highlight the importance of remaining physically active in midlife.

In the current study, a history of minor trauma fractures in women was identified as a baseline risk factor for admission or death. Minor trauma fractures are typically associated with osteoporosis which can affect both men and women, but women are at higher risk due to oestrogen withdrawal during menopause. Minor trauma fractures are recognised as a leading cause of hospitalization among older adults in the UK [19]. The risk of subsequent fractures rises after an initial fracture, making it critical to identify and manage the initial fracture [20]. This also reduces hospital costs and the burden on the health system [21]. Our data showing that fragility fracture was associated with later hospital admission/death among women, therefore, add support to the urgent call for Fracture Liaison Services [20, 22].

The current study has a number of strengths. First, the data used were collected routinely by the HES service for England. They cover all patients treated in NHS hospitals, and NHS patients treated in private hospitals. Only privately funded patients attending private hospitals and those treated outside England are excluded. Second, HES data have the advantage that they are not subject to the effects of attrition of the sickest members of the cohort. Response bias, which is introduced if the least healthy individuals are selectively lost to follow-up, is a particular problem among ageing cohorts [23]. HES data accrue for all members of the cohort, whether they live independently or not, and irrespective of cognitive ability. Thirdly, continued follow-up of hospital admissions into advanced old age was possible with the new data linkage service launched by NHS Digital to streamline the process. Linkage with the HCS enables individuals who experienced hospital admission and deaths to be compared with those who did not. In summary, we have shown that it is possible, with appropriate consent, to link routinely collected HES data with the detailed information collected as part of a cohort study. Our study does have limitations, however. A healthy responder bias has been described in participants in the HCS [6], and our power to examine associations between some lifestyle risk factors, such as heavy alcohol consumption, and health outcomes may be limited. Our study focuses on inpatient admissions and, therefore, we do not consider outpatient care. Furthermore, the study was conducted in the relatively affluent Southeast of England [24] and participants were White Caucasian so findings may be less generalizable to community-dwelling older people of other ethnic groups or among those living in areas of greater deprivation. However, the demographic, social, and medical characteristics among HCS participants have been shown to be broadly comparable with those in the nationally representative Health Survey for England [25]. Another limitation is that only univariate associations between baseline characteristics and risk of admission/death were examined. However, after adjustment for age and socioeconomic status (social class among men and home ownership status among women), all univariate associations remained significant with the exception of the association between smoking status and risk of admission/death among men (data not shown). This suggests that lifestyle, physical performance and health status are related to risk of admission/death, even after accounting for socioeconomic status.

Our results show that hospital admission patterns have not changed in recent years despite the implementation of multiple evidence-based interventions in clinical and public health sectors. In recent years, several interventions have been introduced to reduce hospital admissions including multifactorial fall prevention programs and lifestyle interventions (social prescribing to promote physical activity, health screenings, and smoking bans) to improve the health and wellbeing of older people. Longer follow-up is now required to assess their effectiveness to improve health further and reduce hospital admissions.

In summary, we have reported updated hospital admission patterns from a community-dwelling cohort of UK adults. We related a range of sociodemographic factors, self-reported measures of health and modifiable lifestyle factors to risk of admission/death. Of the modifiable factors studied (BMI, physical activity, cigarette smoking and alcohol consumption), we saw some differences between men and women. These most likely reflect our power to study associations; for example, the prevalence of cigarette smoking was lower in women. However, we note that the prevalence of admission types varied by sex, with higher risk of falls and fractures among women. There is some evidence that body composition may be differentially associated with falls risk in the two sexes, so it is possible that the sexual dimorphism we observed is real, especially since the mean BMI was similar in the two sexes [26]. We hope these data may assist healthcare providers and those planning services that predict future hospital use. Furthermore, we also identified a cohort of individuals who survived to later life without having hospital admissions, a group that may be important to understand better as we consider how to ‘age better’.

### Supplementary Information

Below is the link to the electronic supplementary material.

Supplementary file1 (DOCX 13 KB)

## Data Availability

Hertfordshire Cohort Study data (excluding mortality and Hospital Episode Statistics data) are accessible via collaboration. Initial enquires should be made to EMD. Potential collaborators will be sent a collaborators’ pack and asked to submit a detailed study proposal to the HCS Steering Group.
