# Supplementary material for: Hospital admissions and mortality over 20 years in community-dwelling older people: findings from the Hertfordshire Cohort Study
Source: Aging Clin Exp Res. 2023 Sep 13;35(11):2751–7. doi: 10.1007/s40520-023-02554-0 (PMC10628036; doi:10.1007/s40520-023-02554-0)
Supplement: Supplementary file 1 — Supplementary file1 (DOCX 13 KB) [file 40520_2023_2554_MOESM1_ESM.docx]

| **Supplementary Table 1: ICD-10 codes used to define types of hospital admission** | |
| --- | --- |
| **Admission type** | **ICD-10 codes** |
| **Neurological** | G00-G99: Diseases of the nervous system |
| **Cardiovascular** | I10-I79 |
| **Myocardial infarction** | I21: Acute myocardial infarction  I22: Subsequent myocardial infarction |
| **Stroke** | I60: Subarachnoid haemorrhage  I61: Intracerebral haemorrhage  I63: Cerebral infarction  I64: Stroke, not specified as haemorrhage or infarction |
| **Respiratory** | J00-J99: Diseases of the respiratory system |
| **Any fracture** | M80: Osteoporosis with pathological fracture  M84: Disorders of continuity of bone  S22: Fracture of rib(s), sternum and thoracic spine  S32: Fracture of lumbar spine and pelvis  S42: Fracture of shoulder and upper arm  S52: Fracture of forearm  S62: Fracture at wrist and hand level  S72: Fracture of femur  S82: Fracture of lower leg, including ankle  S92: Fracture of foot, except ankle  T02: Fractures involving multiple body regions  T08: Fracture of spine, level unspecified  T10: Fracture of upper limb, level unspecified  T12: Fracture of lower limb, level unspecified  M81: Osteoporosis without pathological fracture  M82: Osteoporosis in diseases classified elsewhere  M83: Adult osteomalacia  M90.7: Fracture of bone in neoplastic disease  S02: Fracture of skull and facial bones  S12: Fracture of neck  T90.2: Sequelae of fracture of skull and facial bones  T91.1: Sequelae of fracture of spine  T91.2: Sequelae of other fracture of thorax and pelvis  T92.1: Sequelae of fracture of arm |
|  |  |
| **Hip fracture** | S72.0 Fracture of neck of femur; Fracture of hip NOS  S72.1 Pertrochanteric fracture; intertrochanteric fracture; trochanteric fracture  S72.2 Subtrochanteric fracture |
|  |  |
| **Fall** | W00-W19: Falls |
